# Hyperdense Artery Sign in Patients With Acute Ischemic Stroke–Automated Detection With Artificial Intelligence-Driven Software

**DOI:** 10.3389/fneur.2022.807145

**Published:** 2022-04-05

**Authors:** Charlotte Sabine Weyland, Panagiotis Papanagiotou, Niclas Schmitt, Olivier Joly, Pau Bellot, Yahia Mokli, Peter Arthur Ringleb, A. Kastrup, Markus A. Möhlenbruch, Martin Bendszus, Simon Nagel, Christian Herweh

**Affiliations:** ^1^Department of Neuroradiology, University of Heidelberg, Heidelberg, Germany; ^2^Department of Neuroradiology, Klinikum Bremen-Mitte, Bremen, Germany; ^3^Department of Radiology, Areteion University Hospital, National and Kapodistrian University of Athens, Athens, Greece; ^4^Brainomix Ltd., Oxford, United Kingdom; ^5^Department of Neurology, University of Heidelberg, Heidelberg, Germany; ^6^Neurology, Klinikum Bremen-Mitte, Bremen, Germany

**Keywords:** acute ischemic stroke, computed tomography, artificial intelligence, hyperdense artery sign, large vessel occlusion

## Abstract

**Background:**

Hyperdense artery sign (HAS) on non-contrast CT (NCCT) can indicate a large vessel occlusion (LVO) in patients with acute ischemic stroke. HAS detection belongs to routine reporting in patients with acute stroke and can help to identify patients in whom LVO is not initially suspected. We sought to evaluate automated HAS detection by commercial software and compared its performance to that of trained physicians against a reference standard.

**Methods:**

Non-contrast CT scans from 154 patients with and without LVO proven by CT angiography (CTA) were independently rated for HAS by two blinded neuroradiologists and an AI-driven algorithm (Brainomix®). Sensitivity and specificity were analyzed for the clinicians and the software. As a secondary analysis, the clot length was automatically calculated by the software and compared with the length manually outlined on CTA images as the reference standard.

**Results:**

Among 154 patients, 84 (54.5%) had CTA-proven LVO. HAS on the correct side was detected with a sensitivity and specificity of 0.77 (CI:0.66–0.85) and 0.87 (0.77–0.94), 0.8 (0.69–0.88) and 0.97 (0.89–0.99), and 0.93 (0.84–0.97) and 0.71 (0.59–0.81) by the software and readers 1 and 2, respectively. The automated estimation of the thrombus length was in moderate agreement with the CTA-based reference standard [intraclass correlation coefficient (ICC) 0.73].

**Conclusion:**

Automated detection of HAS and estimation of thrombus length on NCCT by the tested software is feasible with a sensitivity and specificity comparable to that of trained neuroradiologists.

## Introduction

Large vessel occlusion (LVO) is the underlying cause in about 30% of patients with acute ischemic stroke (AIS) (1, 2). Since endovascular treatment (EVT) was established as a standard treatment for LVO in the anterior circulation, the identification of eligible patients has become the foremost priority. This led to an increase in CT angiography (CTA) examinations (3). Recently, it was shown that the systematic increase of CTAs in patients with AIS also results in an increased number of identified LVOs and hence an increase of EVTs (4). However, it was recently shown that up to 20% of LVOs might be initially missed on CTA depending on the degree of training and specialization of the reader (5). Furthermore, CTA might still be withheld in AIS, especially in primary stroke centers or smaller hospitals, or because of worries for side effects of contrast agent, e.g., in case of kidney disease or possible allergic reactions. Finally, AIS may not be initially suspected at all and thus missed, either in the prehospital setting which might result in misrouting the patient, or in the emergency department which occurs in 9–26% (6–8). This might occur especially in mildly affected patients, i.e., with an NIHSS below five which is reported in up to 12% of LVO cases (9). Currently, an ongoing clinical trial investigates the effects of EVT in less severely affected patients (10).

In patients with acute LVO, a hyperdense artery sign (HAS) in the localization of the occlusion may be seen on non-contrast CT-imaging (NCCT) (11). This can be further differentiated into HAS typically in the M1 segment and the so-called middle cerebral artery (MCA) dot sign, which refers to more peripheral occlusions in Sylvian MCA branches running orthogonally to the image plane (12). HAS has been shown to correlate with the histological composition of emboli as density increases with the portion of red blood cells (RBC) (13–15). On the other hand, fibrin-rich emboli are indicative of cardio embolism as etiology (14, 16). Recently, it could be shown that the composition of emboli and hence their potential source can be derived from artificial intelligence (AI)-assisted analysis of CT images (17). Besides indicating potential stroke etiology, HAS prevalence can also be used to predict the success of EVT with hyperdense, i.e., RBC-rich clots being less reluctant to thrombectomy than less dense, i.e., fibrin-rich clots of potential cardiac origin (18–20).

Therefore, detection of HAS can be beneficial in two ways, not only by predicting potential EVT success but also by accelerating the initiation of EVT. This is especially when patients are being referred from smaller hospitals to comprehensive stroke centers. Accordingly, it was shown recently that HAS, together with clinical information, reached an accuracy of 91% for LVO detection prior to referral in a “drip-and-ship” scenario (21).

Artificial intelligence is being increasingly employed to assist and accelerate image interpretation, especially in stroke CT-imaging. Initially, the detection of early ischemic changes by determining the Alberta Stroke Program Early CT Score (ASPECTS) was shown to benefit from this approach (22), while automated LVO detection on CTA was also proven reliable recently (23, 24).

We aimed to evaluate an algorithm based on AI which identifies HAS depending on the presence of an LVO against the CTA-based reference standard and compare its performance to that of specialized physicians.

## Methods

### Patients

Computed tomography examinations from 154 non-consecutive patients treated in three different centers for acute stroke or stroke-like symptoms were selected for this study. Among these patients, 84 (54.5%) had AIS due to LVO, i.e., occlusions of the intracranial internal carotid artery (ICA), as well as proximal MCA (M1 and proximal M2, respectively). On the other hand, 70 patients presented with minor stroke symptoms or mimics without LVO. In each center, there was a general approval from the local Institutional Review Board (IRB) for retrospective collection and analysis of clinical and imaging data from stroke patients, with informed consent being waived and under which the data for the present study were collected.

### Imaging

For broad applicability, CT scans each comprising NCCT and CTA were selected from three different sites, and five different scanners from three different vendors were used, which were the following: three 64-row scanners (SOMATOM Definition AS, Siemens Healthineers, Germany; Ingenuity Core, Philips, Netherlands and Aquilion 64, Toshiba) and two 16-row scanners (Emotion 16, Siemens Healthineers, Germany and MX16, Philips, Netherlands). All examinations comprised standard quality NCCTs and CTA with a slice thickness of 1 mm or less for both NCCT and CTA.

A board-certified neuroradiologist with 18 years of experience reviewed the CTA scans for the presence and exact location of an occlusion defining the reference standard. NCCT images were independently analyzed for the presence and side of a HAS by two neuroradiology residents with 2 and 3 years of experience, respectively. These specialists were unaware of the CTA results or any clinical information. Additionally, the length of occlusion was determined on CTA images manually by one of the neuroradiologists (reader 1) after being unblinded.

### Deep Learning Algorithm

**Figure 1:**
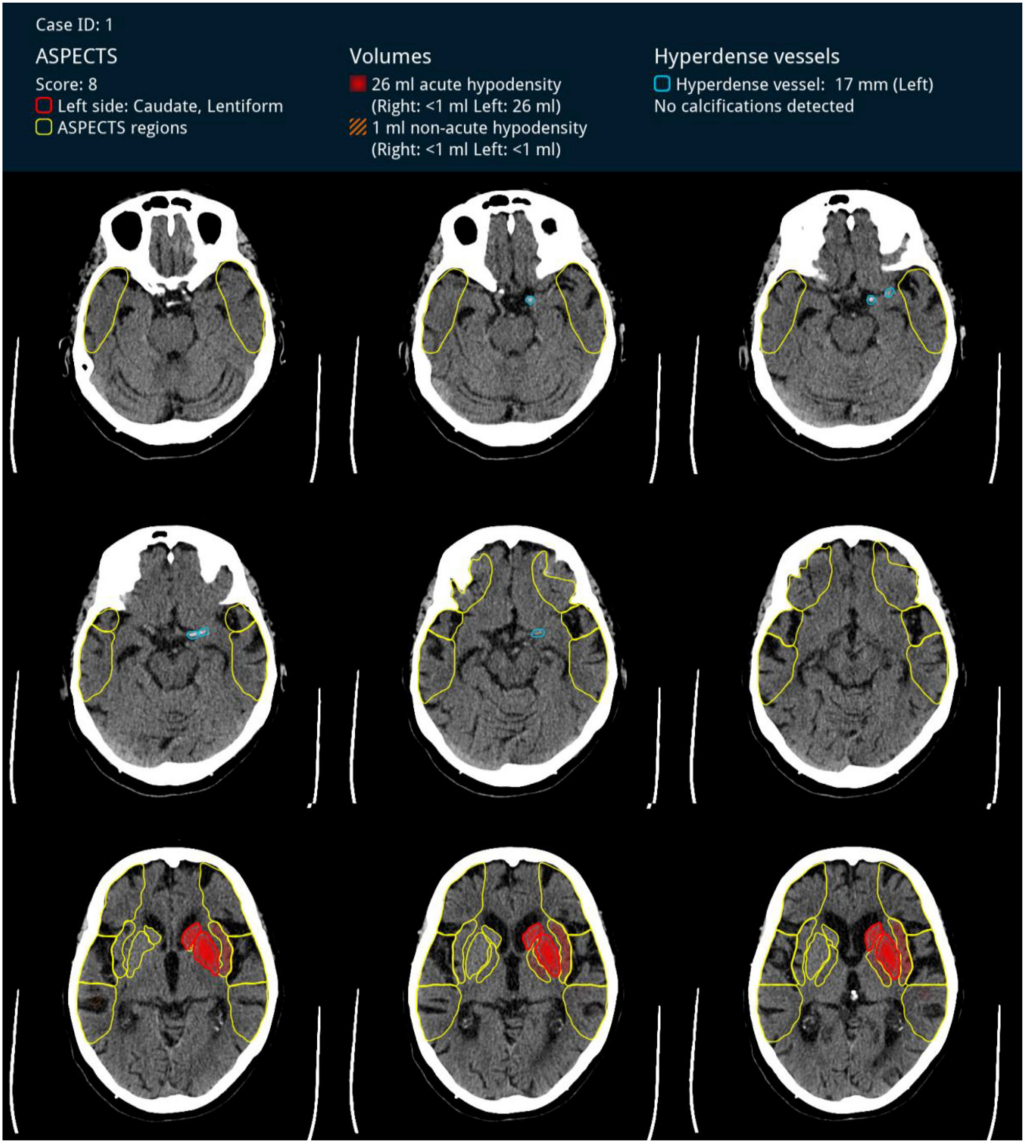
Visualization of the software output—hyperdense artery sign (encircled in light blue) in the M1 segment together with early ischemic changes in the caudate and lentiform nucleus (marked in red) in a patient with acute M1-occlusion on the left side.

All NCCT images were also automatically analyzed by an algorithm developed by Brainomix® (Oxford, UK) based on a three-dimensional fully convolutional neural network (CNN) that has demonstrated its utility in a variety of automated image classification and segmentation tasks (25). The CNN is designed to follow structures starting from the level of the circle of Willis up to the level of early MCA branches in the Sylvian fissure. The model is learning from both hemispheres to better distinguish HAS from generally hyperdense vessels (e.g., due to an elevated hematocrit). Besides the incidence of a HAS, its length is also automatically measured (see Figure 1). The CNN had previously been trained on a dataset of more than 500 cases with 55% harboring an LVO. The present analysis was carried out for a single operating point considered most appropriate within a clinical context.

### Statistical Analysis

Sensitivity and specificity, as well as the agreement with the reference standard (Cohen's kappa) (11) with regard to presence and identification of the correct side, was calculated for the software and each reader. A receiver-operating-curve (ROC) analysis was also carried out. The intraclass correlation coefficient (ICC; model: alpha; type: absolute agreement; two-way-mixed model) (26) was calculated to analyze the agreement between the automatically and the manually determined HAS and is reported for a single measurement. The level of significance was set to *p* = 0.05. Statistical analysis was performed using SPSS Version 27 (IBM, USA).

## Results

Patients' median age was 76 years (CI: 42–92) and those with LVO were significantly older than those without (median 70 ± 12.1 vs. 67 ± 19.1 years; *p* = 0.005; Student's *t*-test). Overall, 63 were women and sex distribution did not differ significantly between patients with (52% women) and without LVO (42% women; *p* = 0.29; chi-square). In 84 patients with LVO, the median National Institutes of Health Stroke Scale (NIHSS) score was 15 (CI: 4–24). Additionally, 76 patients were treated with massage therapy (MT) of which 43 had intravenous tissue plasminogen activator (IV tPA). Furthermore, 8 patients had suspected arterio-arterial embolism due to cervical artery stenosis proximal to the LVO. Other risk factors in these patients were arterial hypertension in 76 (90%), atrial fibrillation in 41 (48%), and diabetes in 10 (12%). Data were missing for 23 patients of which five had an LVO. See Table 1 for a synopsis of demographic and clinical data.

**Table 1:** Demographic and clinical data.

**Baseline clinical data**	**LVO (84)**	**Non-LVO (70)**	***p*-Value**
Sex (female; 63/154)	39.1% (9)	54.5% (24)	0.30^X^
Age, mean +/-SD	75 ± 12.1	67 ± 19.1	0.005^#^^*^
NIHSS on admission, median (range)	15 (4–24)	NA	
Hypertension	90% (76)	NA	
Diabetes	12% (10)	NA	
Atrial fibrillation	48% (41)	NA	
Cervical artery stenosis	9% (8)	NA	
Mechanical thrombectomy	90% (76)	NA	
Intravenous thrombolysis	51% (43)	NA	

χ *Chi-square-test*;

# *Student's t-test*;

* *Significant*.

*NA, not available. Cardiovascular risk factors, as well as NIHSS scores, are not given for patients without LVO since this group comprised several patients who had CT and CTA for other reasons, i.e., transient neurological symptoms, seizure, or trauma*.

**Figure 2:**
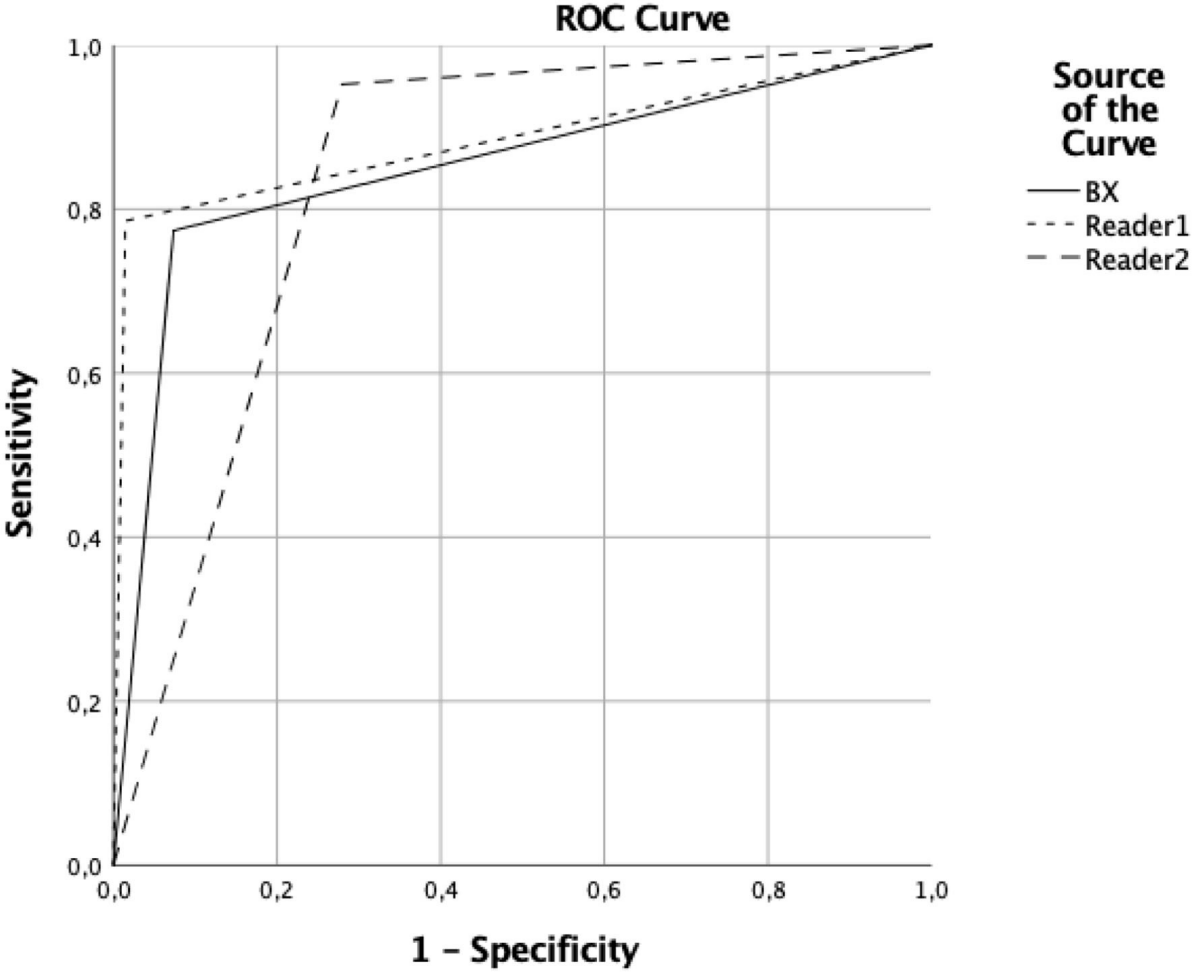
Receiver operating curves (ROC) for the software and 2 human readers ROC curves showing the accuracy of detecting hyperdense artery signs by the software (BX) and two human readers (Readers 1 and 2).

In 84 patients with LVO proven by CTA, occlusions were in the distal ICA in 31, in the M1 segment in 49, with an overlap between these locations in three, and in the M2 segment in seven cases. HAS was detected by the software on the correct side with a sensitivity and specificity of 0.77 (0.66–0.85) and 0.87 (0.77–0.94), respectively, as well as 0.8 (0.69–0.88) and 0.97 (0.89–0.99) by reader 1 and 0.93 (0.84–0.97) and 0.71 (0.59–0.81) by reader 2, respectively (see Figure 2). Agreement with the CTA-based reference standard was 0.71, 0.8, and 0.75, respectively. The ROC analysis yielded similar areas under the curve (AUC) values as well (see Table 1). The median clot length was 15 mm on CTA (IQR: 12–22) and the ICC for the automatically determined HAS length was 0.73 (0.5–0.84). See Table 2 for a survey of the results.

**Table 2:** Accuracy for correct detection of hyperdense artery sign on the correct side for all occlusions as well as ICA and MCA (M1 and 2) occlusions separately.

		**Sensitivity**	**Specificity**	**PPV**	**NPV**	AUC^*^
**BX HAS**	All occlusons (*n* = 84)	0.77 (0.66–0.85)	0.87 (0.77–0.94)	0.87 (0.77–0.94)	0.77 (0.66–0.85)	0.85 (0.79–0.91)
	ICA (*n* = 31)	0.78 (0.59–0.9)	0.9 (0.79–0.95)	0.78 (0.59–0.9)	0.9 (0.79–0.95)	
	MCA (*n* = 53)	0.72 (0.57–0.82)	0.9 (0.79–0.95)	0.84 (0.69–0.93)	0.81 (0.69–0.88)	
**Reader 1**	All occlusions (*n* = 84)	0.8 (0.69–0.88)	0.97 (0.89–0.99)	0.97 (0.88–0.99)	0.8 (0.69–0.87)	0.88 (0.82–0.94)
	ICA (*n* = 31)	0.93 (0.77–0.98)	0.97 (0.89–0.99)	0.93 (0.77–0.98)	0.97 (0.89–0.99)	
	MCA (*n* = 53)	0.71 (0.57–0.82)	0.97 (0.89–0.99)	0.95 (0.82–0.99)	0.82 (0.72–0.89)	
**Reader 2**	All occlusions (*n* = 84)	0.93 (0.84–0.97)	0.71 (0.59–0.81)	0.79 (0.70–0.86)	0.89 (0.77–0.95)	0.83 (0.76–0.90)
	ICA (*n* = 31)	0.94 (0.77–0.98)	0.71 (0.59–0.81)	0.6 (0.45–0.73)	0.96 (0.85–0.99)	
	MCA (*n* = 53)	0.94 (0.83–0.98)	0.71 (0.59–0.81)	0.71 (0.59–0.81)	0.94 (0.83–0.98)	

*NPV, negative predictive value; PPV, positive predictive value; confidence intervals are provided in brackets*;

* *not side-specific*.

In 21 cases, the software did not detect a HAS in presence of an LVO, in which 9 and 3 of those were not detected by readers 1 and 2, respectively. These false-negative results could be attributed to target vessel occlusions either very distal, i.e., in the M2 segment (*n* = 7) or very proximal, i.e., in the carotid terminus (*n* = 5). Among the remaining cases, five showed additional atherosclerosis, in which one was given a contrast agent previously. In 7 cases, the software detected a HAS in absence of an LVO, in which 1 and 5 were also erroneously rated positive by readers 1 and 2, respectively. This could be attributed either to focal atherosclerosis or prior administration of contrast agent in 2 cases, respectively. In 10 of these 21 (47%) false-negative cases, atrial fibrillation was the assumed etiology of LVO.

## Discussion

In the present study, automated detection of HAS by an AI-driven software application on NCCT images from patients with AIS due to LVO showed performance similar to trained physicians. There was a moderate agreement between the automatically determined absolute length of the occlusion and the length manually outlined on CTA images as reference.

There are a few reports so far on automated detection of HAS in acute LVO, reporting similar results see Table 3. A recently published study analyzed the performance of different commercial software (Methinks LVO®) in 1,453 consecutive patients with AIS, among which 823 had LVO in different locations, including the posterior circulation (27). The authors report values for sensitivity and specificity of 83.2 and 71.3%, respectively, and report that the performance could be improved by adding clinical information. Automated detection was better in proximal occlusions in the anterior circulation. Thrombus length was also automatically assessed but without reference standard. Similar results were presented by another group for the Viz HDVS® software (28). In this study, where 117 out of 223 patients had an LVO in the M1 segment, sensitivity and specificity were 70 and 96%, respectively. It should be noted that in these two studies, the performance of the particular software was compared to the reference standard only, i.e., the presence of an LVO on CTA. However, the detection of a HAS by a human reader very much depends on the individual skills and level of training. Previous studies reported poor and also a good interrater agreement with κ-values between 0.59 (11) and 0.91 (12) for HAS cases with angiography-proven LVO. At the same time, the prevalence of the hyperdense artery sign in the anterior circulation is reported with considerable variation. In patients with AIS attributable to the MCA territory but without proven occlusion, i.e., no angiography or CTA, the prevalence of the HAS is reported between 5 (30) and 41% (31). In a large metanalysis in patients with proven M1 occlusion, 405 of 768 patients had a HAS yielding a sensitivity and specificity for the presence of an LVO of 52.4 and 94.9%, respectively (11). The authors also found increased sensitivity (62%) of the HAS with thinner NCCT slices. Despite proven LVO, the aforementioned variability of HAS might be caused by different clot origins and hence compositions. Since these affect their density on CT images with cardioembolic fibrin-rich clots being more lucent than RBC-rich clots (14, 16). In the present study, the rate of patients with atrial fibrillation was rather high with 48% given the fact that the prevalence of atrial fibrillation in AIS patients is reported to be around 25% in general (32), as well as in LVO patients (33). This relatively higher percentage of potentially more lucent emboli of cardiac origin might therefore account for false-negative HAS findings in the present study cohort. Therefore, when validating an automated HAS detection, it is mandatory to have comparative readings by physicians to better characterize the imaging data of the study cohort. Using exclusively slices at a thickness of 1 mm in the present study, sensitivity and specificity of two blinded neuroradiologists were 80 and 97%, as well as 93 and 71%, respectively. This is in keeping with results from another study comparing performances of human readers with different slice thicknesses (34) where sensitivity (73%) and specificity (83%) were best with 1 mm slices and worsened with increasing slices thickness. Comparing AI and human readers, very similar results were presented in 2019 by a group using another commercial application (Nico.lab®) in 59 patients with proven LVO out of 107 (29). They found a sensitivity and specificity for the software of respective 86 and 65%, while for two expert readers results were respective 95 and 58%, as well as 79 and 82%. It is of note that in both studies one of two readers had high sensitivity and low specificity while this relation was inverted for the other reader. It is likely that less trained or specialized readers would perform poorer which would increase the potential benefit of such software. Nevertheless, it should not be considered to replace CTA in AIS with suspected LVO rather than to flag cases in which LVO is not suspected or CTA may be withheld for other reasons.

**Table 3:** Comparison of different hyperdense artery sign detection algorithms introduced so far in the literature (March 2021).

**Sensitivity/ Specificity**	**Brainomix® (84/154)**	**MethinksLVO® (27) (823/1,453)**	**Viz HDVS® (28) (117/223)**	**Nico.lab® (29) (59/107)**
Software	0.77/0.87	0.82/0.71	0.70/0.96	0.86/0.65
Reader 1	0.80/0.97			0.79/0.82
Reader 2	0.93/0.71			0.95/0.58

In the present study, NCCT images from 5 different scanner models from 3 different vendors were used. This is notable since most vendors provide different proprietary image reconstruction algorithms, which can affect automated image analyses (35). Therefore, the performances of the software and readers can be considered independent of such scanner-specific features and the results as representative.

The length of an LVO can be relevant for therapeutic decision-making. In the present study, the length of the occlusion automatically determined by the software on NCCT images was in moderate to good agreement with the CTA-based reference standard. In the aforementioned studies, only one reported automated length measurements but without providing reference standards (27).

### Limitations

The present study was designed to prove feasibility and the results must be considered preliminary. It suffered from typical shortcomings of the retrospective design, i.e., lack of control of the data collected. Furthermore, clinical data was missing for a few cases for which there was only imaging data. The data was from non-consecutive cases from several centers and there was a clear selection bias since the cases were actively chosen to comprise comparable numbers of patients with and without LVO, ignoring the actual prevalence in real life i.e., 23% in the anterior circulation AIS (2). On this basis, the positive predictive value of the software would decline to 0.64 while that of readers 1 and 2 would change to 0.88 and 0.48, respectively. Furthermore, M2 occlusions were underrepresented and the performance of the software at this location could not be properly estimated based on the present results. Moreover, the prevalence of atrial fibrillation was increased as well in our cohort, and LVO patients were significantly older than those without. Also, the study group was rather small. Therefore, the present results must be interpreted with caution and could not be readily translated into real life. We tried to compensate for that by reporting clinical data, where available, showing that the NIHSS and age of LVO patients in the present cohort were within a range typically reported in studies on AIS. Furthermore, we only used CT images at a slice thickness of 1 mm to achieve the best performance of human readers and the software too. The results are not reproducable with thicker slices especially in older CT-scanners.

## Conclusion

In this feasibility study, the AI-driven Brainomix® software performed similarly to neuroradiology residents in detecting HAS in case of an acute LVO in the proximal anterior circulation. These results are preliminary and need to be confirmed in real-life consecutive cohorts. If so, this might help to trigger and prime further investigation in patients in whom LVO was not suspected primarily.

## Data Availability Statement

The raw data supporting the conclusions of this article will be made available by the authors, without undue reservation.

## Ethics Statement

The studies involving human participants were reviewed and approved by Ethikkommission Med. Fakultät Heidelberg. Written informed consent for participation was not required for this study in accordance with the national legislation and the institutional requirements.

## Author Contributions

OJ, PB, PP, and CH contributed to conception and design of the study. CW, NS, SN, and CH organized the database. CH performed the statistical analysis. CW and CH wrote the first draft of the manuscript. OJ, PB, PP, and MB wrote sections of the manuscript. All authors contributed to manuscript revision, read, and approved the submitted version.

## Conflict of Interest

MB: Unrelated: grants from Siemens, Stryker, Hopp foundation, grants, and personal fees from Novartis and Guerbet, personal fees from Merck, Teva, Grifols, BBraun, Boehringer Ingelheim, Vascular Dynamics, Springer, Bayer, all outside the submitted work. MM: Unrelated: Consultancy: Medtronic, MicroVention, Stryker; Payment for Lectures Including Service on Speakers Bureaus: Medtronic, MicroVention, Stryker. ^*^Money paid to the institution. OJ: Head of the Scientific Research at Brainomix, Oxford, UK. PB: Deep learning researcher at Brainomix, Oxford, UK. PR: Unrelated: Consultancy: Boehringer, Lecture fees from Bayer, Boehringer Ingelheim, BMS, Daichii Sankyo, Pfizer. SN: Unrelated: Consultancy: Brainomix, Boehringer Ingelheim; Payment for Lectures Including Service on Speakers Bureaus: Pfizer, Medtronic, Bayer AG. CH: Related: Consultancy: Brainomix, Oxford, UK. OJ and PB were employed by Brainomix Ltd. The remaining authors declare that the research was conducted in the absence of any commercial or financial relationships that could be construed as a potential conflict of interest.

## Publisher's Note

All claims expressed in this article are solely those of the authors and do not necessarily represent those of their affiliated organizations, or those of the publisher, the editors and the reviewers. Any product that may be evaluated in this article, or claim that may be made by its manufacturer, is not guaranteed or endorsed by the publisher.

## Abbreviations

AI: artificial intelligence
AIS: acute ischemic stroke
CNN: convolution neural network
EVT: endovascular therapy
HAS: hyperdense artery sign
LVO: large vessel occlusion
MCA: middle cerebral artery
NCCT: non-contrast Computer Tomography.
