# Supplementary material for: Hyperdense Artery Sign in Patients With Acute Ischemic Stroke–Automated Detection With Artificial Intelligence-Driven Software
Source: Front Neurol. 2022 Apr 5;13:807145. doi: 10.3389/fneur.2022.807145 (PMC9016329; doi:10.3389/fneur.2022.807145)
Supplement: Supplementary file 1 [file Table_1.docx]

|  | | **occlusion in CTA** | |
| --- | --- | --- | --- |
|  |  | **present** | **absent** |
| **BX HAS** | occlusion detected | 62 | 5 |
|  | no occlusion detected | 22 | 65 |
| **Reader 1** | occlusion detected | 66 | 2 |
|  | no occlusion detected | 18 | 68 |
| **Reader 2** | occlusion detected | 78 | 20 |
|  | no occlusion detected | 6 | 50 |
| total | | 84 | 70 |

**Supplemental table – Raw, individual results for general LVO**
